# Within-household SARS-CoV-2 transmission and vaccine effectiveness in the first three COVID-19 school outbreaks in northern Viet Nam, September–December 2021

**DOI:** 10.5365/wpsar.2024.15.3.1077

**Published:** 2024-07-11

**Authors:** Trang Thu Vu, Tu Huy Ngo, Khanh Cong Nguyen, Vu Thi Lan, Cu Thi Bich Hanh, Le Hong Son, Huyen Thi Nguyen, Hien Thi Nguyen, Nghia Duy Ngu, Duong Nhu Tran, Duc-Anh Dang, Florian Vogt, Thai Quang Pham

**Affiliations:** a National Centre for Epidemiology and Population Health, College of Health and Medicine, Australian National University, Canberra, Australian Capital Territory, Australia.; b Department of Communicable Disease Control and Prevention, National Institute of Hygiene and Epidemiology, Hanoi, Viet Nam.; c Field Epidemiology Training Program, National Institute of Hygiene and Epidemiology, Hanoi, Viet Nam.; d Ha Nam Center for Disease Control, Ha Nam, Viet Nam.; e Phu Tho Center for Disease Control, Phu Tho, Viet Nam.; f Thanh Hoa Center for Disease Control, Thanh Hoa, Viet Nam.; g National Institute of Hygiene and Epidemiology, Hanoi, Viet Nam.; h The Kirby Institute, University of New South Wales, Sydney, New South Wales, Australia.; i Department of Research Methodology and Biostatistics, School of Preventive Medicine and Public Health, Hanoi Medical University, Hanoi, Viet Nam.; * These authors contributed equally to this work as shared first authors.; ** These authors contributed equally to this work as shared last authors.

## Abstract

**Objective:**

The risk of transmission of severe acute respiratory syndrome coronavirus 2 (SARS-CoV-2) from schoolchildren to their household and the protective effects of vaccination in these settings remain poorly understood. We assessed the transmission dynamics of schoolchildren with SARS-CoV-2 within their households and the protective effects of coronavirus disease (COVID-19) vaccination among household members in Viet Nam.

**Methods:**

We estimated the attack rate, vaccine effectiveness and adjusted risk ratio (aRR) of factors associated with SARS-CoV-2 transmission to household contacts of children confirmed to have COVID-19 who attended three schools in Ha Nam, Phu Tho and Thanh Hoa provinces between September and December 2021 using multivariable regression with household-level random effects.

**Results:**

This retrospective cohort study included 157 children infected with SARS-CoV-2 and their 540 household contacts. The attack rate among household contacts was 24.6% (133/540). Overall, vaccine effectiveness among household contacts was 39% (95% confidence interval [CI]: −1 to −63), higher among males than females and higher in adults aged > 40 years. COVID-19 transmission was greater among female household contacts compared with males (aRR: 1.35, 95% CI: 0.94 to 1.95), although not statistically significant, and highest among those aged 19–39 years (aRR: 2.51, 95% CI: 1.50 to 4.21). Fully vaccinated household contacts had significantly lower infection risk (aRR: 0.46, 95% CI: 0.26 to 0.84).

**Discussion:**

We found substantial onward transmission of SARS-CoV-2 from schoolchildren to household members, and older people were more likely to be protected by vaccination. We recommend that schoolchildren and all household members living with schoolchildren receive at least two doses of a COVID-19 vaccine. Recognizing the role of schoolchildren in the onward transmission of COVID-19 is an important lesson learned by Viet Nam that can help not only in managing other outbreaks but also in protecting schoolchildren by predicting the progress of the outbreak and preparing for a timely response.

On 23 January 2020, Viet Nam announced its initial cases of coronavirus disease (COVID-19). These were quickly followed by sporadic clusters of imported and secondary cases, which subsequently transformed into clusters in the community in late March and April 2020. Public health measures were implemented across the country, including widespread social isolation, the requirement for all visitors and repatriated people to be tested for severe acute respiratory syndrome coronavirus 2 (SARS-CoV-2) and placed in quarantine, the implementation of intensive contact tracing and school closures, and restrictions on intercity transportation. After the final community cases were identified in late April 2021, these public health and social measures were gradually stopped in May 2021. From May until July 2021, only imported cases were detected through quarantine and immigration checkpoints, and no special public health and social measures, such as national lockdown or school closures, were carried out. (*1*)

In 2021, Viet Nam used emergency regulations to approve nine COVID-19 vaccines: ChAdOx1-S [recombinant] vaccine (AstraZeneca), Abdala (CIGB-66, Center for Genetic Engineering and Biotechnology), Spikevax (Moderna), Sputnik V (Gamaleya Research Institute), CoronaVac vero cell vaccine (Sinovac Life Sciences), Comirnaty (Pfizer–BioNTech), Hayat-Vax (Sinopharm), Janssen (Johnson & Johnson) and Covaxin (Bharat Biotech). Most of the vaccine doses were supplied by AstraZeneca, Gamaleya Research Institute, Sinovac Life Sciences, Pfizer–BioNTech and Moderna. Viet Nam began COVID-19 vaccinations for adults in April 2021.

From September to November 2021, several regions reopened their schools, after which several school outbreaks were caused by the SARS-CoV-2 Delta strain. Ha Nam, Phu Tho and Thanh Hoa provinces, which had reported no COVID-19 cases previously, all reported school outbreaks. Because the vaccination programme targeted only adults, all children younger than 18 years were unvaccinated. The Ministry of Health began vaccinating children aged 12–17 years against COVID-19 on 14 October 2021. Children aged 12–17 years were vaccinated in Ha Nam on 16 November 2021, in Phu Tho on 14 November 2021 and in Thanh Hoa on 30 November 2021.

The role of children in school outbreaks and within-household transmission of COVID-19 is not well understood. One study from England reported that children are less likely to transmit the illness than adults, (*2*) with another study from Ireland reporting no secondary transmission within a school setting. (*3*) An unpublished study from United Kingdom of Great Britain and Northern Ireland found that children aged 11–18 years had the highest rate of COVID-19, (*4*) and those aged 5–11 years had a prevalence comparable to working-age people (Riley S, Ainslie KEC, Eales O, Walters CE, Wang H, Atchison C, et al. High prevalence of SARS-CoV-2 swab positivity and increasing R number in England during October 2020: REACT-1 round 6 interim report. medRxiv [Preprint]. 2020). In 2023, a study from United States of America that included 110 children indicated that they could carry and spread the virus at rates similar to adults. (*5*) Moreover, a 2022 systematic review found lower prevalence in children compared with adults, but these rates increased with the arrival of new variants such as Omicron. (*6*)

Unvaccinated children infected by SARS-CoV-2 are frequently asymptomatic or have minimal symptoms; therefore, their role in disease transmission within households must be considered. Several studies have shown that transmission rates of SARS-CoV-2 within a household were higher than transmission in schools. (*7*-*10*) However, the role of vaccination status among household members has not yet been documented. Addressing these gaps will allow for a more evidence-based approach to school closures and vaccine prioritization strategies.

In this study, we aimed to assess within-household attack rates and the effect of vaccination among household members living with unvaccinated children with confirmed SARS-CoV-2 infection during three school outbreaks in Ha Nam, Phu Tho and Thanh Hoa provinces, Viet Nam, between September and December 2021.

## Methods

### Study design and sampling

We used secondary data collected during three school outbreak investigations in Ha Nam, Phu Tho and Thanh Hoa. Data were available for 157 students and 540 household contacts from the three outbreaks: 23 students and 87 household contacts in Ha Nam; 91 students and 347 household contacts in Phu Tho; and 43 students and 106 household contacts in Thanh Hoa.

### Case definition

A confirmed case was defined as a child who tested positive for SARS-CoV-2 by real-time reverse transcription–polymerase chain reaction (RT–PCR) and who attended one of the three implicated schools. A household contact was defined as anyone who lived in the same household as a confirmed case at the time of recruitment. When a schoolchild exhibited symptoms or was a contact of another confirmed case, the child and all household members underwent RT–PCR testing. Household members were tested regardless of symptoms. Household members who tested negative at the time the confirmed case was diagnosed and also tested negative after a 14-day period of self-quarantine were considered not to be cases.

### Study variables

#### Outcome variable

The outcome variable was the COVID-19 test result of household members. Vaccine effectiveness was calculated using the formula: VE = (1 - RR) × 100, where RR indicates the attack rate of fully vaccinated people divided by the attack rate of those who were unvaccinated or received only one dose.

#### Independent variables

The demographic information for confirmed cases included age, sex (male/female) and whether they had any symptoms at the time of testing (yes/no). Symptoms were classified as yes or no for fever, cough, sneezing, fatigue, headache or abdominal pain, loss of taste or smell and trouble breathing. Age was then categorized as preschool, primary school, secondary school and high school.

We obtained demographic information for household members from data collected during the outbreak investigation, including age, sex (male/female), vaccination status (fully vaccinated, defined as having received at least two vaccination doses, or not vaccinated) and relationship to the student who was a confirmed case (parent, sibling, grandparent or uncle). The ages of household members were divided into three groups: 0–18, 19–39 and ≥ 40 years. The vaccines received were classified into five types: none, only AstraZeneca, only Moderna or Pfizer–BioNTech (mRNA vaccine), only CoronaVac vero cell vaccine, or a mix of two types of vaccine. Cycle threshold (Ct) values for positive schoolchildren and positive household contacts were categorized as < 20, 20 to < 25, 25 to < 30 and ≥ 30.

### Data management

Schoolchildren whose records lacked information about household contacts were excluded from the analysis, as were students residing in the same household as a confirmed case, contacts who were not household members, and household members whose COVID-19 test results were missing (Supplementary Fig. 1). Information collected from the secondary data source was confirmed with the provincial Center for Disease Control and the school, as needed.



### Statistical analysis

Frequencies and percentages were used for descriptive analyses to characterize confirmed cases and their household contacts. The attack rate for household contacts, which equalled the secondary attack rate for confirmed cases among schoolchildren, was calculated by dividing the number of household members with a positive COVID-19 result by the total number of people in the household. This attack rate was then divided into four groups: 0, 0 to 0.5, 0.5 to 1 and 1. The reproduction number was calculated by multiplying the attack rate with the number of household contacts of each index case.

To identify factors associated with household contacts becoming infected with SARS-CoV-2, χ^2^ tests and a univariate regression analysis with mixed effects were used to select potential variables for the multivariable analysis. Variables with *P* < 0.1 in the univariate analysis were eligible for the multivariable mixed effects regression analysis, whereas variables with *P* < 0.05 were considered significant factors associated with COVID-19 infection among household members.

The effectiveness of vaccination against COVID-19 was assessed by comparing vaccinated household members with those who had not been vaccinated. Stata 16 was used for both descriptive and analytical statistics (StataCorp, College Station, TX, USA).

## Results

### Confirmed cases

**Fig. 1:**
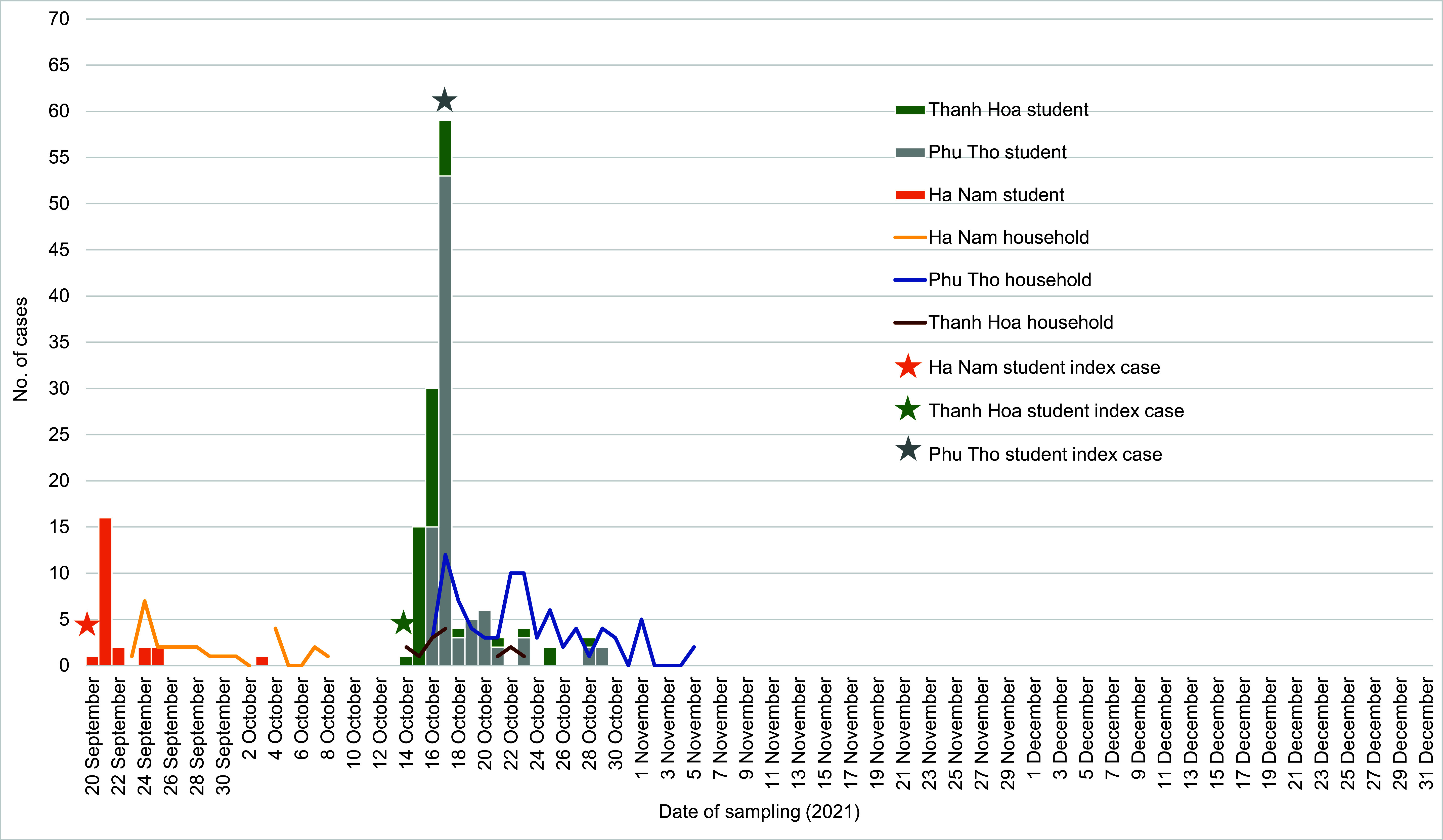
Epidemic curve of students and household members with COVID-19 infection, by date of sampling, in Ha Nam, Phu Tho and Thanh Hoa provinces, Viet Nam, September to December 2021

There were a total of 157 confirmed COVID-19 cases in the school outbreaks in the three provinces. Ha Nam reported its first case from the affected school on 20 September 2021. Case numbers increased the next day. High numbers of household members tested positive for COVID-19 on 21 and 24 September. The outbreak in Phu Tho was reported on 16 October 2021, with cases rapidly increasing on 17 October. Thanh Hoa reported its first confirmed case on 14 October, and from 14 to 25 October, several household members were affected (**Fig. 1**).

[**insert** Figure 1]

The median (interquartile range [IQR]) age for cases at each school in Ha Nam was 13 years (13 to 13 years; only seventh grade students were infected), in Phu Tho it was 13 years (13 to 14 years) and in Thanh Hoa it was 8 years (8 to 11 years). There were more female than male cases in Phu Tho (53% [48/91] and 47% [43/91], respectively), but male cases outnumbered female cases in both Ha Nam (65% [15/23] and 35% [8/23], respectively) and Thanh Hoa (58% [25/43] and 42% [18/43], respectively). The proportion of cases with Ct values < 20 was 74% (17/23) in Ha Nam, 31% (28/91) in Phu Tho and 35% (15/43) in Thanh Hoa. The proportion of cases that were symptomatic was 70% (16/23) in Ha Nam, 53% (48/91) in Phu Tho and 14% (6/43) in Thanh Hoa. Fever was the most common symptom reported by confirmed cases: 61% (14/23) in Ha Nam, 42% (38/91) in Phu Tho and 12% (5/43) in Thanh Hoa. In Ha Nam, 48% (11/23) of cases reported having a loss of taste or smell; for Phu Tho, the proportion was 4% (4/91) and for Thanh Hoa, it was 0% (0/43) (**Table 1**).

**Table 1:** Characteristics of COVID-19-positive students in Ha Nam, Phu Tho and Thanh Hoa provinces, Viet Nam, September to December 2021

Factor	Province^a^		
	Phu Tho	Ha Nam	Thanh Hoa
**Total**	**91**	**23**	**43**
**Age group (years)**			
**Preschool (0–5)**	**0 (0)**	**0 (0)**	**1 (2)**
**Primary school (6–10)**	**0 (0)**	**0 (0)**	**31 (72)**
**Secondary school (11–14)**	**81 (89)**	**23 (100)**	**2 (5)**
**High school (15–18)**	**10 (11)**	**0 (0)**	**9 (21)**
**Median (IQR)**	**13 (13–14)**	**13 (13–13)**	**8 (8–11)**
**Sex**			
**Male**	**43 (47)**	**15 (65)**	**25 (58)**
**Female**	**48 (53)**	**8 (35)**	**18 (42)**
**Ct value of infected students**			
** < 20**	**28 (31)**	**17 (74)**	**15 (35)**
**20 to < 25**	**26 (29)**	**3 (13)**	**13 (30)**
**25 to < 30**	**16 (18)**	**2 (9)**	**6 (14)**
** ≥ 30**	**21 (23)**	**1 (4)**	**9 (21)**
**Median (IQR)**	**23.0 (19.0–29.0)**	**15.3 (12.2–20.5)**	**22.2 (17.5–29.8)**
**Any symptom at time of testing**			
**No**	**43 (47)**	**7 (30)**	**37 (86)**
**Yes**	**48 (53)**	**16 (70)**	**6 (14)**
**Fever**			
**No**	**53 (58)**	**9 (39)**	**38 (88)**
**Yes**	**38 (42)**	**14 (61)**	**5 (12)**
**Cough**			
**No**	**78 (86)**	**17 (74)**	**40 (93)**
**Yes**	**13 (14)**	**6 (26)**	**3 (7)**
**Sneeze**			
**No**	**90 (99)**	**19 (83)**	**40 (93)**
**Yes**	**1 (1)**	**4 (17)**	**3 (7)**
**Fatigue**			
**No**	**91 (100)**	**17 (74)**	**38 (88)**
**Yes**	**0 (0)**	**6 (26)**	**5 (12)**
**Headache or abdominal pain**			
**No**	**90 (99)**	**22 (96)**	**43 (100)**
**Yes**	**1 (1)**	**1 (4)**	**0 (0)**
**Loss of taste or smell**			
**No**	**87 (96)**	**12 (52)**	**43 (100)**
**Yes**	**4 (4)**	**11 (48)**	**0 (0)**
**Trouble breathing**			
**No**	**90 (99)**	**23 (100)**	**43 (100)**
**Yes**	**1 (1)**	**0 (0)**	**0 (0)**
**Household contacts**			
**Median no. (IQR)**	**4 (3–4)**	**4 (3–4)**	**2 (2–3)**
**Attack rate in household**			
**0**	**34 (37)**	**11 (48)**	**33 (77)**
** > 0 to < 0.5**	**40 (44)**	**0 (0)**	**2 (5)**
**0.5 to < 1**	**11 (12)**	**7 (30)**	**1 (2)**
**1**	**6 (7)**	**5 (22)**	**7 (16)**
**Median (IQR)**	**0.2 (0–0.33)**	**0.5 (0–0.75)**	**0 (0–0)**

Ct: cycle threshold value; IQR: interquartile range.

^a^ Values are *n*(%) unless other wise indicated.

### Household contacts

There were 540 household contacts included in this study. Of these, 133 were positive for COVID-19, giving an overall attack rate among all household contacts of 24.6%. The attack rate was highest in Ha Nam at 37% (32/87), followed by 24% (83/347) in Phu Tho and 17% (18/106) in Thanh Hoa. The median (IQR) Ct values among household members who tested positive for COVID-19 were 16.6 (13.3 to 26.7) in Ha Nam, 20 (17 to 27) in Phu Tho and 21.9 (19.1 to 31.4) in Thanh Hoa. Confirmed cases had an overall reproduction number of 0.85.

In Ha Nam and Phu Tho, almost half of household members who tested positive for COVID-19 were aged ≥ 40 years (47% [15/32] and 42% [35/83], respectively). In Thanh Hoa, 28% (5/18) were aged ≥ 40 years, with more than half (67% [12/18]) aged 19–39 years. The proportion of household members with COVID-19 who were female was 50% (16/32) in Ha Nam, 60% (50/83) in Phu Tho and 67% (12/18) in Thanh Hoa (**Table 2**).

**Table 2:** Demographic information for household members living with schoolchild who tested positive for COVID-19 in Ha Nam, Phu Tho and Thanh Hoa provinces, Viet Nam, September to December 2021

Demographic information	Province^a^								
	Phu Tho			Ha Nam			Thanh Hoa		
**Factor^b^**	**Positive**	**Negative**	**Total**	**Positive**	**Negative**	**Total**	**Positive**	**Negative**	**Total**
**SARS-CoV-2**	**83**	**264**	**347**	**32**	**55**	**87**	**18**	**93**	**106**
**Age group (years)**									
**0–18**	**14 (17)**	**88 (33)**	**102 (29)**	**9 (28)**	**17 (31)**	**26 (30)**	**1 (6)**	**21 (24)**	**22 (21)**
**19–39**	**34 (41)**	**57 (22)**	**91 (26)**	**8 (25)**	**12 (22)**	**20 (23)**	**12 (67)**	**32 (36)**	**44 (42)**
** ≥ 40**	**35 (42)**	**115 (44)**	**150 (43)**	**15 (47)**	**26 (47)**	**41 (47)**	**5 (28)**	**34 (39)**	**39 (37)**
**Median (IQR)**	**38 (15–49)**			**39 (16–46)**			**34.5 (24–47)**		
**Sex**									
**Male**	**33 (40)**	**136 (52)**	**169 (49)**	**16 (50)**	**28 (51)**	**44 (51)**	**6 (33)**	**51 (58)**	**57 (54)**
**Female**	**50 (60)**	**128 (48)**	**178 (51)**	**16 (50)**	**27 (49)**	**43 (49)**	**12 (67)**	**37 (42)**	**49 (46)**
**Fully vaccinated (received at least two doses)**									
**No**	**71 (86)**	**198 (75)**	**269 (78)**	**30 (94)**	**48 (87)**	**78 (90)**	**18 (100)**	**88 (100)**	**106 (100)**
**Yes**	**12 (14)**	**66 (25)**	**78 (22)**	**2 (6)**	**7 (13)**	**9 (10)**	**0 (0)**	**0 (0)**	**0 (0)**
**Type of vaccine**									
**None**	**33 (40)**	**131 (50)**	**164 (47)**	**20 (62)**	**27 (49)**	**47 (54)**	**17 (94)**	**87 (99)**	**104 (98)**
**Only AstraZeneca**	**18 (22)**	**34 (13)**	**52 (15)**	**9 (28)**	**22 (40)**	**31 (36)**	**1 (6)**	**1 (1)**	**2 (2)**
**Only Moderna or ** ** Pfizer–BioNTech (mRNA vaccine)**	**7 (8)**	**31 (12)**	**38 (11)**	**2 (6)**	**6 (11)**	**8 (9)**	**0 (0)**	**0 (0)**	**0 (0)**
**Only Sinovac Life Sciences ** ** (vero cell vaccine)**	**22 (27)**	**49 (19)**	**71 (20)**	**0 (0)**	**3 (5)**	**0 (0)**	**0 (0)**	**0 (0)**	**0 (0)**
**Mixed vaccine types**	**2 (2)**	**11 (4)**	**13 (4)**	**0 (0)**	**0 (0)**	**0 (0)**	**0 (0)**	**0 (0)**	**0 (0)**
**Relationship to confirmed case**									
**Parent**	**60 (72)**	**0 (0)**	**60 (17)**	**14 (44)**	**23 (42)**	**37 (43)**	**12 (67)**	**46 (52)**	**58 (55)**
**Sibling**	**16 (19)**	**2 (1)**	**18 (5)**	**12 (38)**	**15 (27)**	**27 (31)**	**2 (11)**	**23 (26)**	**25 (24)**
**Grandparent/uncle**	**7 (8)**	**1 (< 1)**	**8 (2)**	**6 (19)**	**17 (31)**	**23 (26)**	**4 (22)**	**19 (22)**	**23 (22)**
**Ct value**									
** < 20**	**40 (48)**	**–**	**40 (48)**	**16 (50)**	**–**	**16 (50)**	**6 (33)**	**–**	**6 (6)**
**20 to < 25**	**16 (19)**	**–**	**16 (19)**	**2 (6)**	**–**	**2 (6)**	**6 (33)**	**–**	**6 (6)**
**25 to < 30**	**13 (16)**	**–**	**13 (16)**	**6 (19)**	**–**	**6 (19)**	**0 (0)**	**–**	**0 (0)**
** ≥ 30**	**13 (16)**	**–**	**13 (16)**	**3 (9)**	**–**	**3 (9)**	**5 (28)**	**–**	**5 (5)**
**Median (IQR)**	**20 (17–27)**			**16.6 (13.3–26.7)**			**21.91 (19.14–31.43)**		
**Any symptom at time of testing?**									
**No**	**28 (34)**	**2 (1)**	**30 (9)**	**–**	**–**	**–**	**–**	**–**	**–**
**Yes**	**54 (65)**	**16 (6)**	**70 (20)**	**–**	**–**	**–**	**–**	**–**	**–**

Ct: cycle threshold value; IQR: interquartile range.

^a^ Values are *n*(%) unless otherwise indicated; – indicates data are not applicable.

^b^ Some factors do not add up to the total due to missing data.

Regarding vaccination status, 14% (12/83) of household contacts in Phu Tho who tested positive were fully vaccinated, compared with 6% (2/32) of those who tested positive in Ha Nam and 0% in Thanh Hoa (**Table 2**). Higher proportions of household contacts who tested positive were unvaccinated in Ha Nam and Thanh Hoa (62% [20/32] and 94% [17/18], respectively). The majority of vaccinated household members who had COVID-19 had received only the AstraZeneca vaccine: 28% (9/32) in Ha Nam, 22% (18/83) in Phu Tho and 6% (1/18) in Thanh Hoa.

The relationship between confirmed cases and household contacts who tested positive for COVID-19 was also investigated, with 44% (14/32) in Ha Nam, 72% (60/83) in Phu Tho and 67% (12/18) in Thanh Hoa being parents of a confirmed case. The proportions of household members testing positive for COVID-19 who had other family relationships with the case were much lower (**Table 2**).

### Factors associated with COVID-19 infection of household contacts

When comparing household members who tested positive for COVID-19 with household members who were negative for COVID-19 in the multivariable analysis, the risk of being positive for COVID-19 was greater among household contacts aged 19–39 years (adjusted risk ratio [aRR]: 2.51, 95% CI: 1.50–4.21). Household contacts who were fully vaccinated and those who lived with a confirmed case aged 6–10 years had a lower risk of becoming a COVID-19 case (for contacts who were fully vaccinated – aRR: 0.46, 95% CI: 0.26–0.84; for contacts who lived with a confirmed case aged 6–10 years – aRR: 0.15, 95% CI: 0.02–0.93). All other variables were not significant in the multivariable analysis (**Table 3**).

**Table 3:** Univariate and multivariate analysis of selected factors related to transmission among students and household members, Viet Nam, September to December 2021

Variable	SARS-CoV-2-positive^a^	SARS-CoV-2-negative^a^	Univariate			Multivariate^c^	
			aRR	*P^b^*	95% CI	aRR	95% CI
**Household contacts**							
**Sex**							
**Male**	**55 (20.4)**	**215 (79.6)**	**1**	**0.022**	**–**	**1**	**–**
**Female**	**78 (28.9)**	**192 (71.1)**	**1.5**		**1.05–2.13**	**1.35**	**0.94–1.95**
**Fully vaccinated (received at least two vaccination doses)**							
**No**	**119 (26.3)**	**334 (73.7)**	**1**	**0.044**	**–**	**1**	**–**
**Yes**	**14 (16.1)**	**73 (83.9)**	**0.596**		**0.33–1.06**	**0.46**	**0.26–0.84**
**Age group (years)**							
**0–18**	**24 (16.0)**	**126 (84.0)**	**1**	**0.001**	**–**	**1**	**–**
**19–39**	**54 (34.8)**	**101 (65.2)**	**2.17**		**1.33–3.54**	**2.51**	**1.50–4.21**
** ≥ 40**	**55 (23.9)**	**175 (76.1)**	**1.47**		**0.90–2.40**	**1.56**	**0.95–2.56**
**Confirmed cases (students)**							
**Any symptoms at time of testing**							
**No**	**57 (20.3)**	**224 (79.7)**	**1**	**0.053**	**–**	**1**	**–**
**Yes**	**76 (29.3)**	**183 (70.7)**	**1.49**		**0.99–2.22**	**1.02**	**0.50–2.06**
**Fever**							
**No**	**73 (21.7)**	**264 (78.3)**	**1**	**0.039**	**–**	**1**	**–**
**Yes**	**60 (29.6)**	**143 (70.4)**	**1.37**		**0.92–2.05**	**1.05**	**0.54–2.05**
**Cough**							
**No**	**103 (21.9)**	**367 (78.1)**	**1**	** < 0.001**	**–**	**1**	**–**
**Yes**	**30 (42.9)**	**40 (57.1)**	**1.991**		**1.22–3.25**	**1.78**	**0.98–3.24**
**Sneezing**							
**No**	**73 (21.7)**	**264 (78.3)**	**1**	**0.001**	**–**	**1**	**–**
**Yes**	**14 (50.0)**	**14 (50.0)**	**2.268**		**1.12–4.60**	**1.77**	**0.68–4.61**
**Fatigue**							
**No**	**116 (23.0)**	**389 (77.0)**	**1**	**0.001**	**–**	**1**	**–**
**Yes**	**17 (48.6)**	**18 (51.4)**	**2.134**		**1.14–4.00**	**1.22**	**0.46–3.20**
**Age group (years)**							
**Preschool (0–5)**	**2 (100.0)**	**0 (0.0)**	**1**	**0.010**	**–**	**1**	**–**
**Primary school (6–10)**	**12 (15.4)**	**66 (84.6)**	**0.14**		**0.02–0.97**	**0.15**	**0.02–0.93**
**Secondary school (11–14)**	**108 (26.7)**	**296 (73.3)**	**0.258**		**0.041–1.62**	**0.38**	**0.07–2.21**
**High school (15–18)**	**11 (19.6)**	**45 (80.4)**	**0.176**		**0.03–1.23**	**0.28**	**0.04–1.82**

95% CI: 95% confidence interval; aRR: adjusted risk ratio.

^a^ Values are *n*(%) unless otherwise indicated; – indicates data are not applicable.

^b^ *P*-values were calculated using the χ^2^ test.

^c^ All variables with *P* ≤ 0.1 in the univariate analysis were included in the multivariate analysis.

### Vaccine effectiveness for household contacts

The vaccine effectiveness for fully vaccinated household contacts was 39% (95% CI: −1–63; **Table 4**). For male household contacts, vaccine effectiveness was 52% (95% CI: −25–82) and for females it was 34% (95%  CI: −19–63), although this was not statistically significant. The vaccine effectiveness for household contacts differed by age group: 23% (95% CI: −40–58) among those aged 19–39 years and 65% (95% CI: 17–85) among those aged > 40 years. Vaccine effectiveness was not applicable to children aged 0–18 years because they were not eligible for vaccination in Viet Nam when this study was conducted.

**Table 4:** Effectiveness of COVID-19 vaccine among household contacts of schoolchildren who tested positive for COVID-19, Viet Nam, September to December 2021

Factor	Vaccination doses						RR (95% CI)	Vaccine effectiveness^a^ (95% CI)	*P*
	None or one			Two					
	Total	COVID-19-positive	Attack rate (a)	Total	COVID-19-positive	Attack rate (b)			
Total no.	453	119	0.26	87	14	0.16	0.61 (0.37–1.01)	39 (−1–63)	0.044
Age group (years)									
< 18	148	24	0.16	2	0	0.00	–	–	–
18–39	123	45	0.37	32	9	0.28	0.77 (0.42–1.40)	23 (−40–58)	0.313
40–59	179	50	0.28	51	5	0.10	0.35 (0.15–0.83)	65 (17–85)	
Sex									
Male	232	51	0.22	38	4	0.11	0.48 (0.18–1.25)	52 (−25–82)	0.566
Female	221	68	0.31	49	10	0.20	0.66 (0.37–1.19)	34 (−19–63)	

95% CI: 95% confidence interval; RR: relative risk (b/a); – indicates that children < 18 years were not vaccinated during the study period.

**^a^** Vaccine effectiveness is calculated as 1 - RR.

## Discussion

This study assessed the within-household risk of transmission of SARS-CoV-2 from schoolchildren to household members during three school outbreaks in northern Viet Nam occurring between September and December 2021. The overall attack rate for household members of confirmed cases from the schools was 24.6%, lower than in studies from Republic of Korea and Thailand. (*11*, *12*) This disparity could be attributed to the study period, the circulating variant, and differences in countries and populations. Moreover, prior research has also found that the Delta variant had a lower attack rate than the Omicron variant. (*11*)

The secondary attack rate for schoolchildren was 37% in Ha Nam, 24% in Phu Tho and 17% in Thanh Hoa. The median age of confirmed cases among schoolchildren was 13 years in Ha Nam, 13 years in Phu Tho and 8 years in Thanh Hoa. The discrepancy in median age among provinces may account for the lower secondary attack rate in Thanh Hoa, as older children (10–19 years) reportedly had a higher transmission rate than younger children (< 10 years). (*13*) Research by Madewell et al. also found that the secondary attack rate increased for households with more than three people. (*14*) Consequently, the data support our conclusion that the attack rate was higher in Ha Nam and Phu Tho, where the average number of household members per family was four, than in Thanh Hoa, where it was two. Phu Tho and Thanh Hoa had lower secondary attack rates than similar clusters in Peru (53%) (*15*) and China (32.4%). (*16*) The secondary attack rate among household members found recently in China, Republic of Korea and United States of America ranged from 4.6% to 17%.**^17_19^** These discrepancies may be due to each study's sample size, the social distancing that was in place and different quarantine measures among the countries at the time of investigation. Our results showed that factors directly related to the infection rate among household contacts were their sex, age, vaccination status and age of the index case.

Our findings suggest that female household contacts might be more susceptible to contracting infection, although this finding did not reach statistical significance in our data. Our results differ from studies conducted in Norway and Pakistan, where male household members had a higher incidence of infection. (*17*, *18*) However, our findings are consistent with those of researchers in Malaysia and Türkiye who also found that female household contacts had a significantly increased risk for COVID-19. (*19*, *20*) This contrast may result from cultural and social differences between the Vietnamese population and those in other countries.

This study showed that household contacts who were positive for COVID-19 were more likely to be aged 19–39 years, compared with those who were negative for COVID-19. This is consistent with studies from China, Japan, Malaysia and Pakistan. (*17*, *19*, *21*, *22*) A survey conducted in Bosnia and Herzegovina also found that household contacts aged 18–49 years were nearly five times more likely than those aged 0–17 years to become infected. (*23*)

Fully vaccinated household members had a lower risk of becoming ill with COVID-19, consistent with a previous Norwegian study that indicated full COVID-19 vaccination was a protective factor among household contacts. (*18*) Our findings demonstrated a vaccine effectiveness rate of 39% against SARS-CoV-2 infection among household members. This was a lower vaccine effectiveness rate than reported in Singapore (56.4%), (*24*) which may be because only mRNA vaccines were authorized for use in Singapore at the time.

Our findings suggest that vaccine effectiveness may be lower in females than males; although this difference was not statistically significant in our analysis, it is similar to findings in a study in Angola. (*25*) However, the target population in the study in Angola was the general population, not household contacts. Sex differences in caregivers’ duties and activities might also have played a role. (*26*) Additionally, antibody responses following COVID-19 immunization and their duration, as well as the type of vaccine, may impact the vaccine's effectiveness. (*27*, *28*) Therefore, further studies should be considered to clarify this difference. However, there was no statistically significant difference by sex when the analysis was adjusted for age.

There was a significant association between the confirmed case being aged 6–10 years and having household members who tested positive for COVID-19. A cohort study conducted in England found that children younger than 11 years had a lower rate of COVID-19 transmission. (*29*) Our results, however, found that young children (aged 0–5) are more likely to transmit SARS-CoV-2 than older ones, which is consistent with prior research. (*29*, *30*)

The risk of household transmission has been reported to be higher if the child index case is symptomatic, although this finding was not statistically significant. (*31*) Our findings indicate that household contacts of schoolchildren with cough at the time of testing had a higher probability of testing positive for COVID-19. This result is consistent with previous studies that showed coughing by the index case was a significant risk (*17*, *23*) for transmission and an indicator of the index case's infectiousness. (*32*) Furthermore, according to Miller et al., (*29*) index cases without respiratory symptoms had a lower risk of transmitting SARS-CoV-2, which is consistent with our finding that household contacts of students with sneeze or fatigue were at higher risk of becoming infected.

This study's strength lies in using outbreak data to assess factors associated with index cases and their household contacts that related to the role of children transmitting COVID-19 during school outbreaks. However, this study is not without its limitations. First, this study exclusively focused on cases associated with the Delta variant, which is no longer the predominant COVID-19 variant. However, the Delta variant has been reported to have the second-highest transmissibility after Omicron, (*33*) so the results still provide valuable insights for public health workers assessing potential similarities with future COVID-19 variants.

Second, this was an analysis of secondary data, and the routine nature of the data collected led to shortcomings in sampling and testing (e.g. symptoms at the time household contacts were tested may have been missing, as well as the date of symptom onset; serology data were lacking; data were missing about the exact date of testing for contacts). Otherwise, the World Health Organization’s protocols for household transmission studies would have been applied. (*34*) Additionally, many household contacts were unvaccinated; therefore, it was impossible to analyse the protection rate of the vaccines by dose. We could not categorize the vaccination status variable by time because we did not have the date on which household contacts received their COVID-19 vaccine.

Third, this study collected data for many variables, but not data about household contacts’ potential community exposure or self-protection measures. These data would have helped assess independent risk factors. At the time of our study, the three provinces did not have strict lockdowns in place, so we cannot rule out transmission occurring outside the households.

## Conclusions

SARS-CoV-2 transmission from children to their household members was linked with variables associated with the primary case and their household contacts. We found that when the confirmed case was aged 6–10 years and household contacts were unvaccinated and aged 19–39 years, the household members were at lower risk of COVID-19 infection. While the COVID-19 vaccines were primarily developed to reduce disease severity rather than transmission, this study found an overall vaccine effectiveness against infection of 39%. These results should be explored in future studies to improve the Viet Nam health system's preparedness and response capabilities for future pandemics. We strongly recommend that schoolchildren, a vulnerable population, should be prioritized for COVID-19 vaccination. Our attack rates may also provide guidance for future decision-making about school closures by policy-makers.
