# Supplementary material for: Within-household SARS-CoV-2 transmission and vaccine effectiveness in the first three COVID-19 school outbreaks in northern Viet Nam, September–December 2021
Source: Western Pac Surveill Response J. 2024 Jul 11;15(3):1–12. doi: 10.5365/wpsar.2024.15.3.1077 (PMC11304045; doi:10.5365/wpsar.2024.15.3.1077)

Supplementary Fig. 1. **Data cleaning process for study of COVID-19 transmission among schoolchildren and household members, Viet Nam, September to December 2021**

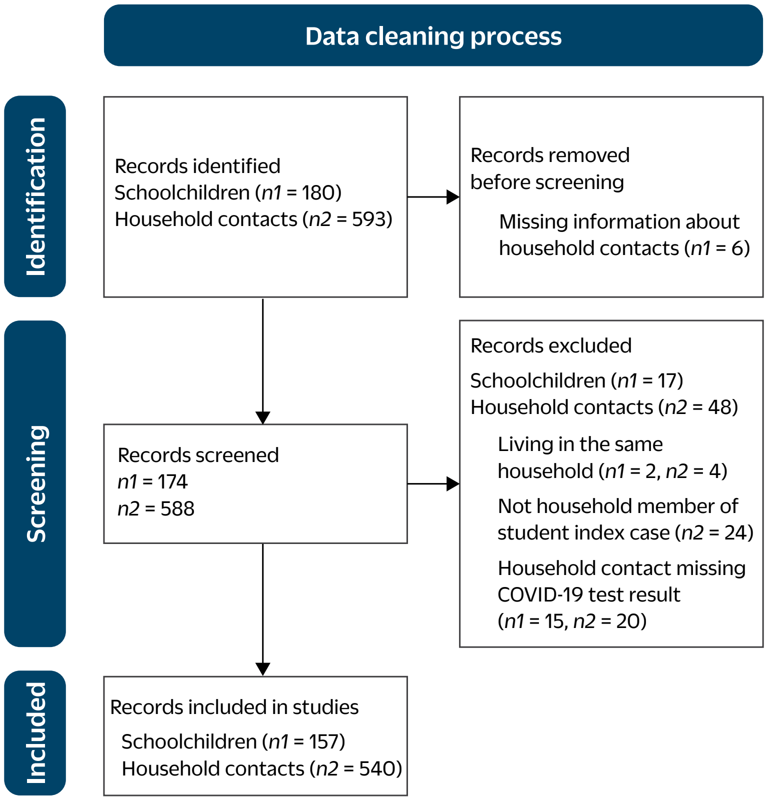

Supplement: Supplementary file 1 [file wpsar-15-1077-s001.pdf]
